# Non-reciprocal robotic metamaterials

**DOI:** 10.1038/s41467-019-12599-3

**Published:** 2019-10-10

**Authors:** Martin Brandenbourger, Xander Locsin, Edan Lerner, Corentin Coulais

**Affiliations:** Institute of Physics, Universiteit van Amsterdam, Science Park 904, 1098 XH Amsterdam, The Netherlands

**Keywords:** Mechanical engineering, Structural materials, Metamaterials, Applied physics

## Abstract

Non-reciprocal transmission of motion is potentially highly beneficial to a wide range of applications, ranging from wave guiding to shock and vibration damping and energy harvesting. To date, large levels of non-reciprocity have been realized using broken spatial or temporal symmetries, yet mostly in the vicinity of resonances, bandgaps or using nonlinearities, thereby non-reciprocal transmission remains limited to narrow ranges of frequencies or input magnitudes and sensitive to attenuation. Here, we create a robotic mechanical metamaterials wherein we use local control loops to break reciprocity at the level of the interactions between the unit cells. We show theoretically and experimentally that first-of-their-kind spatially asymmetric standing waves at all frequencies and unidirectionally amplified propagating waves emerge. These findings realize the mechanical analogue of the non-Hermitian skin effect. They significantly advance the field of active metamaterials for non hermitian physics and open avenues to channel mechanical energy in unprecedented ways.

## Abstract (web summary)

Here, the authors demonstrate a robotic metamaterial implemented through a combination of actuators, sensors and local controllers. They show that this active metamaterial can exhibit tunable linear non-reciprocal dynamic characteristics, with a very large and broadband non-reciprocal gain.

## Introduction

Reciprocity is a fundamental property of linear, time-reversal invariant physical systems, entailing that their response functions are symmetrical, namely that signals are transmitted symmetrically between any two points in space^1–3^. In other words, if one sends an electromagnetic, acoustic, or mechanical signal through a material in one direction, one can also send it in the opposite direction. While breaking reciprocity has been a long-standing challenge in electromagnetics, there has been over the last few years an explosion of interest for breaking reciprocity in optical^4–7^ and micro^8^ waves without magnetic fields, and beyond electromagnetism, i.e., in acoustics^9^, quantum systems^10,11^, and mechanics^12,13^, thus creating new tools to engineer a novel generation of devices and materials that guide, damp, or control energy and information. Non-reciprocity has been achieved by using passive structures combining broken spatial symmetries and nonlinearities^13,14^ and using active time-modulated components that break time-reversal symmetry^3,12,15–17^. These strategies have led to large levels of nonreciprocal isolations, but with input magnitudes or input frequencies that are limited to narrow ranges, and are sensitive to attenuation.

Here, inspired by recent developments in robotics^18,19^ and active metamaterials^12,16,17,20–22^, we create a robotic mechanical metamaterial that uses distributed active control to break reciprocity at the level of the interactions between the building blocks themselves. This work builds on the field of active metamaterials, yet with a key new twist: while active metamaterials only have actuating elements, robotic metamaterials include a combination of local sensing, computation, communication, and actuation. As a result, they feature unique wave phenomena, namely asymmetric modes at all frequencies and unidirectional amplification, and in turn realize large, broadband, linear, and self-amplified nonreciprocal transmission of mechanical waves. These findings realize the classical counterpart of the so-called non-Hermitian skin effect^23–28^.

## Results

### Nonreciprocal wave equation

**Fig. 1:**
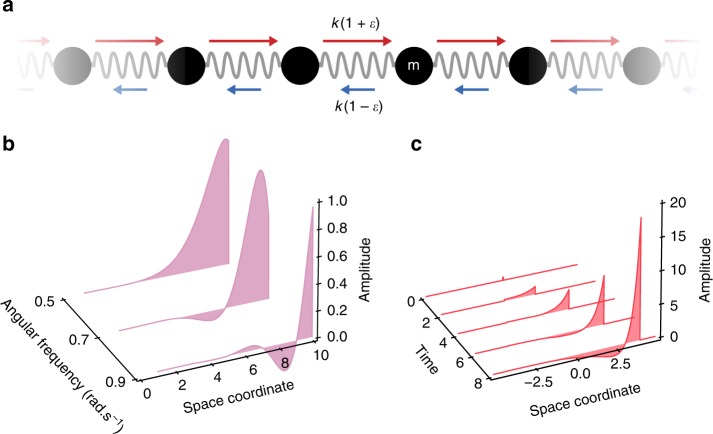
Asymmetric and unidirectionally amplified waves in a nonreciprocal mass-and-spring model. **a** Schematic representation of the nonreciprocal mass-and-spring model. **b** Magnitude of the solutions of Eq. (1) in the frequency domain exp(*i*(*ωt*−*q*_±_*x*)) vs. spatial coordinate, for three different frequencies. **c** Green’s function of Eq. (1) vs. time and spatial coordinate. In (**b**) and (**c**), *ε* = 0.9 and *c* = 0.5

We first investigate theoretically the emergent properties in a mass-and-spring model with nonreciprocal springs (Fig. 1a). For reciprocal mechanical structures^1,29,30^, the stiffness matrix—relating displacements to forces—is symmetrical by virtue of the Maxwell–Betti reciprocity theorem^1^. In particular, for a simple spring, left-to-right and right-to-left stiffnesses are equal: *k*_L→R_ = *k*_R→L_ = *k* where *k*_L→R_ and *k*_R→L_ are defined as *k*_L→R_ = *F*_L→R_/(*u*_R_ − *u*_L_) and *k*_R→L_ = *F*_R→L_/(*u*_L_ − *u*_R_), where *F*_L_ (*F*_R_) is the force on the left (right) spring and *u*_L_ (*u*_R_) the displacement of the left (right) spring. Here, we consider a special mass-and-spring model, where the left-to-right and right-to-left stiffnesses differ $k_{{\mathrm{L}} \to {\mathrm{R}}} = k(1 + \varepsilon ) \ \ne \ k_{{\mathrm{R}} \to {\mathrm{L}}} = k(1 - \varepsilon )$ (Fig. 1a). We obtain the following continuum equation (Methods, Mass-and-spring model with nonreciprocal springs)

1 $$\frac{1}{{c^2}}\frac{{d^2u}}{{dt^2}} - \frac{{d^2u}}{{dx^2}} + \frac{{2\varepsilon }}{p}\frac{{du}}{{dx}} = 0,$$

where $c = p\sqrt {k/m}$ and where *p* is the interparticle distance. In the case of reciprocal interactions (*ε* = 0), Eq. (1) becomes the wave equation, which admits dispersion-free mechanical waves of group and phase velocity *c*. For nonreciprocal interactions (*ε* ≠ 0), the first-order term in Eq. (1) breaks inversion symmetry *u* → −*u*, *x* → −*x*. This asymmetry has dramatic consequences on the nature of the mechanical waves, which can be readily seen from the solutions of this equation both in the frequency domain and in real space. In the frequency domain, solutions consist of a linear combination of the functions exp(*i*(*ωt*−*q*_±_*x*)), where the wave vector $q_ \pm = \frac{i}{p}(\varepsilon \pm \sqrt {\varepsilon ^2 - \omega ^2p^2/c^2} )$. For small frequencies *ω* < *c*|*ε*|/*p*, these solutions are exponentially localized standing waves, while for large frequencies *ω* > *c*|*ε*|/*p*, they are localized oscillatory standing waves with an exponential envelope (Fig. 1b). Crucially, for *ε* > 0 (*ε* < 0), the imaginary part is always positive (negative), so these solutions are always localized on the right (left) edge. In real space, we obtain the Green’s function of Eq. (1) (Methods, Mass-and-spring model with nonreciprocal springs and Supplementary Information Note 1), which is an asymmetric step function propagating at a velocity *c* with a wave front magnitude given by exp(*εct*/*p*)/2 (exp(−*εct*/*p*)/2) for *x* > 0 (*x* < 0). For any value of *ε* > 0 (*ε* < 0), the initial pulse is amplified for forward (backward) propagation and attenuated for backward (forward) propagation (Fig. 1c). This behavior can be intuitively understood from the structure of Eq. (1): work is injected in (extracted from) the wave front when $\frac{{2\varepsilon }}{p}\frac{{du}}{{dx}}$ is negative (positive), whereby the system is constantly driven out-of-equilibrium. This leads to waves with two unprecedented features, namely spatial asymmetry at all frequencies and unidirectional amplification.

### Nonreciprocal robotic metamaterial

**Fig. 2:**
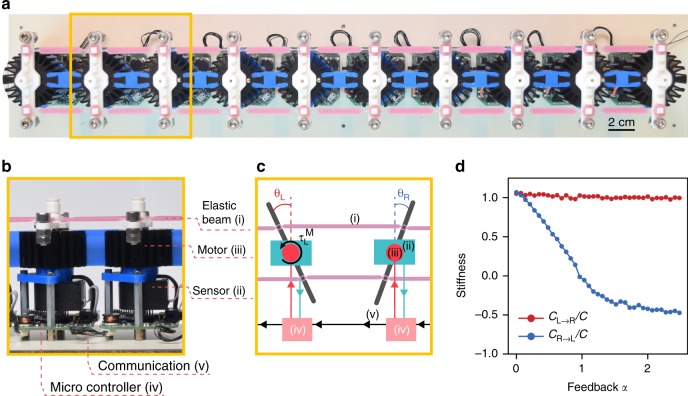
Robotic metamaterial with nonreciprocal interactions. **a** Robotic metamaterial made of 10 unit cells mechanically connected by soft elastic beams (i). Scale bar: 2 cm. (bc) Closeup **b** and sketch **c** on two unit cells. Each unit cell is a minimal robot with a unique rotational degree of freedom that comprises an angular sensor (ii), a coreless DC motor (iii), and a microcontroller (iv). Each unit cell communicates with its right neighbor via electric wires (v). These components allow to program a control loop characterized by the feedback parameter *α* (see main text for definition). **d** Rescaled torsional stiffnesses *C*_L→R_/*C* (red) and *C*_R→L_/*C* (blue) as a function of the feedback parameter

In order to create a system with such effective nonreciprocal local interactions, a necessary but not sufficient condition is to add external forces. Our strategy for achieving such nonreciprocal interactions is to apply strain-dependent forces at each site, i.e., forces that are proportional to the strain in the neighboring springs^22^. These local forces inject—linear or angular—momentum and work into the mechanical degrees of freedom. To do so, we built a metamaterial made of ten “robotic” building blocks (Fig. 2a) with rotational degrees of freedom. Each robotic unit cell consists of a mechanical rotor with a rotational moment of inertia *J*, of a local control system, and is mechanically coupled to its neighbors via pre-stretched elastic beams resulting in a torsional stiffness *C* (Fig. 2b, c). The control system measures the rotor’s angular position *θ*_L_, collects that of its right neighbor *θ*_R_, and applies an additional torque on the left rotor *τ*_M_ = *Cf*(*α*)(*θ*_L_ − *θ*_R_). The parameter *α* is a dimensionless feedback parameter. The feedback gain *f*(*α*) plays a similar role as the parameter *ε* in the model of Fig. 1, yet with a subtle difference. In the experiment, the active force is applied only on the right neighbor, whereas in the model, the active force is applied on both left and right neighbors (Methods, Mass-and-spring model with nonreciprocal springs). We calibrate the torque vs. angle response between two unit cells and find, as expected, that *C*_L→R_ = *C* differs from *C*_R→L_ = *C*(1 − *f*(*α*)) (Fig. 2d), therefore breaking reciprocity. While such tunable nonreciprocal response is not surprising—ultimately it is achieved at the level of each unit cell’s microcontroller—the novelty of our approach lies in coupling many such robotic nonreciprocal unit cells together and making use of the fact that the bandwidth of the electronic components is much larger than that of the mechanical degrees of freedom. As a result of the interaction between multiple robotic building blocks, unique nonreciprocal wave phenomena emerge, as we will see in the following sections.

**Fig. 3:**
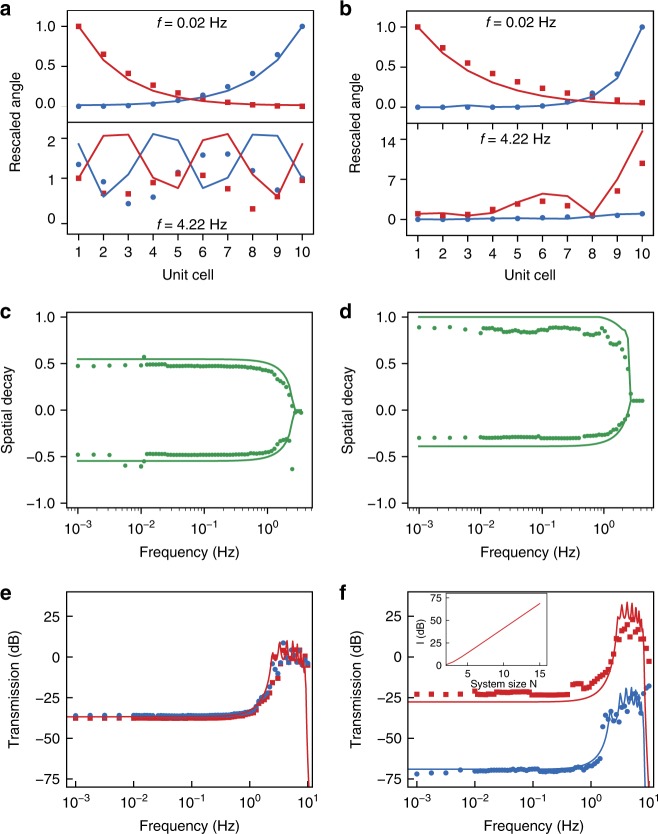
Spatially asymmetric standing waves and broadband unidirectional transmission. Stationary response under sinusoidal excitations at the left (red squares) or right (blue dots) edge of the metamaterial. (ab) Amplitudes of oscillation rescaled by the amplitude of actuation as a function of the unit cell position for sinusoidal excitations with *α* = 0 (**a**) and *α* = 0.43 (**b**) at frequencies 0.02 Hz (top) and 4.22 Hz (bottom). See also Supplementary Video 1. (cd) Spatial decay rates of the amplitudes of oscillation vs. frequency with *α* = 0 (**c**) and *α* = 0.43 (**d**). (ef) Left-to-right (*T*_L→R_) and right-to-left (*T*_R→L_) transmissions as a function of the excitation frequency (f) for a feedback parameter *α* = 0 (**e**) and *α* = 0.43 (**f**). (f-inset) Isolation at low frequency *I* = *T*_L→R_ − *T*_R→L_ as a function of the number of unit cells N, for *α* = 0.43. For each graph, markers depict the experiments and solid lines the numerical model. Note that beyond 10 Hz our experimental system cannot drive and measure

To test the predictions of the mass-and-spring model, we now investigate experimentally and numerically the stationary response of our ten-unit cells robotic metamaterial to harmonic excitations on the left and on the right edges over a wide range of input frequencies (Methods, Calibration and measurements). In the reciprocal case *α* = 0 (Fig. 3a), we observe from experiments that the amplitudes of oscillation of each unit cell either decay exponentially (low frequencies) or oscillate (high frequencies) from one unit cell to another. We model the robotic metamaterial as 10 coupled oscillators interacting with each other via nonreciprocal stiffnesses *C*_L→R_ and *C*_R→L_. To do so, we take into account additional effects such as the bending of the rubber bands and the inherent damping of the oscillators and quantify them via independent calibration (Methods, Calibration and measurements). The model matches the observations very accurately without any fit parameters until 3 Hz, above which the numerical model is too simplistic to accurately capture internal vibrations of the rubber bands (Methods, Numerical model of the robotic metamaterial). For all actuation frequencies, we observe from the model and the experiment that the responses to left and right excitations is simply related to mirror symmetry, which demonstrates that the metamaterial response is inherently symmetrical. In contrast, in the nonreciprocal case *α* = 0.43 (Fig. 3b), we observe a strong asymmetry in the angular displacement profiles. When excited from the right, the response is more localized close to the excitation point and when excited from the left, the response is more extended toward the right and even increases for large frequencies. This asymmetry is further quantified by the spatial decays of the profiles, which are opposite in the reciprocal case *α* = 0 (Fig. 3c) and differ in the nonreciprocal case *α* = 0.43 (Fig. 3d), regardless of the driving frequency. Figure 3d therefore demonstrates the emergence of asymmetric modes at all frequencies, as predicted by the solutions of Eq. (1) and reminiscent of the non-Hermitian skin effect^23–28^.

Does such strong asymmetry lead to nonreciprocal transmission? To address this question, we calculate the transmissions $T_{{\mathrm{L}} \to {\mathrm{R}}} = 20\log _{10}|\bar \theta _{\mathrm{R}}^{{\mathrm{out}}}/\bar \theta _{\mathrm{L}}^{{\mathrm{in}}}|$ and $T_{{\mathrm{R}} \to {\mathrm{L}}} = 20\log _{10}|\bar \theta _{\mathrm{L}}^{{\mathrm{out}}}/\bar \theta _{\mathrm{R}}^{{\mathrm{in}}}|$ for various frequencies from the angular displacement profiles obtained above. In the reciprocal case (*α* = 0), we observe a symmetrical transmission, typical of a resonant low-pass filter, with a saturated plateau of amplitude **−**37 ± 1 dB at small frequencies and a broad peak corresponding to the system resonances beyond which the transmission signal starkly decreases at 360 dB/decade (Fig. 3e). For the nonreciprocal case (*α* = 0.43), the left-to-right and right-to-left transmissions differ vastly—by more than 50 dB—over a wide range of frequencies, from 0.001 to 5 Hz (Fig. 3f). Strikingly, as opposed to previous observations in mechanical metamaterials whose functionality decays with system size^31,32^, such nonreciprocal isolation increases linearly with the system size (Fig. 3f—inset). Therefore, the isolation of the system can be controlled by varying the feedback *α* or by adding more unit cells, the latter having the advantage of avoiding limitations in the maximal torque applied by the control loop. Importantly, our metamaterial is linearly stable over a wide range of feedback parameters (*α* < 0.93) (Methods, Numerical model of the robotic metamaterial). Therefore, the existence of the asymmetric modes at all frequencies leads to an extremely large level of nonreciprocal transmission over a very large range of frequencies, a performance that is unprecedented among wave-based physical systems.

**Fig. 4:**
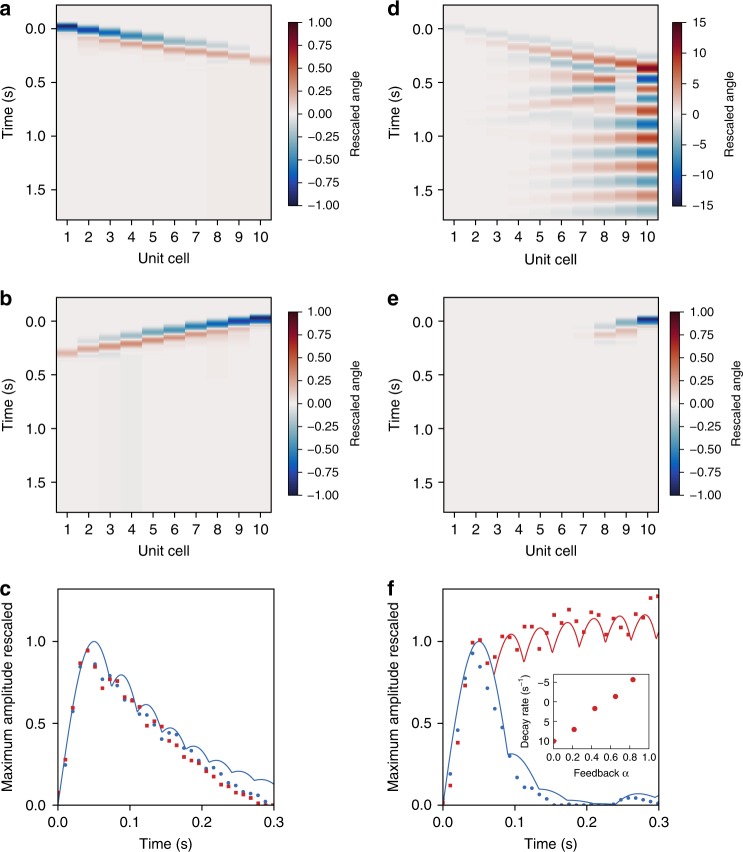
Unidirectional pulse amplification. **a**–**e** Contour plots of the angular displacement vs. space coordinate and time for a feedback parameter *α* = 0 (ab) and *α* = 0.62 (de), upon pulse excitation on the left (ad) and right (be) edge of the metamaterial. (cf) Instantaneous maximum magnitude of the propagation pulse vs. time for a feedback parameter *α* = 0 (c) and *α* = 0.62 (**f**). The points correspond to the experimental data, the thin solid lines to the numerical model without any fit parameters. (f-inset) Decay rate *δ* derived from exponential fits of the form exp(−*δt*) on the maximum amplitude of pulses propagating from left to right for different values of the feedback parameter. See also Supplementary Video 2

Since the nonreciprocal transmission is broadband, our robotic metamaterial is in principle an excellent nonreciprocal device for pulses, which have a broadband spectral signature. To demonstrate this, we excite our metamaterial with half-sine shaped pulses closely mimicking a pulse of amplitude 0.04 rad and duration 100 ms, either from the left or right edge. In the reciprocal case *α* = 0, the response is strictly the same regardless of the excitation point (Fig. 4a–c). The pulses propagate across the metamaterial, reach the opposite edge, and rapidly attenuate upon reflection. After a short transient and before the first reflection, the pulse amplitude decreases (Fig. 4c). By contrast, in the nonreciprocal case *α* = 0.62 (Fig. 4d–f), the pulse attenuates strongly when excited from the right and is amplified when excited from the left. In the latter case, the pulse reaches the right edge of the robotic metamaterial and remains localized in the vicinity of this edge from where it slowly decays in time (see Supplementary Fig. 7). To quantify the pulse attenuation, we restrict our attention to the propagation before the first reflection (Fig. 4c, f). We find that the unidirectional amplification is controlled by the level of feedback *α*: the signal is amplified (attenuated) for *α* > 0.5 (*α* < 0.5) (see Fig. 4f—inset). These observations are in qualitative agreement with the behavior of the Green’s function of Eq. (1) discussed above.

## Discussion

To conclude, we have created a class of robotic mechanical metamaterials that are embedded with nonreciprocal interactions through local control loops. As a result, they feature a unique type of wave phenomena, which show spatial asymmetry of standing waves at all frequencies, leading to an unprecedented broadband giant level of non-reciprocity, and which exhibit unidirectional amplification of pulses. As opposed to existing metamaterials by using nonlinearities or active components, where the nonreciprocal effects tend to be suppressed by attenuation for large system sizes^31^, the key new feature here is that nonreciprocal waves are unidirectionally amplified, which guarantees robustness against attenuation. Nonreciprocal robotic metamaterials could be used to extract mechanical energy, allowing energy to flow away from a source while preventing energy to flow back to it. Therefore, we envision that further developments on nonreciprocal robotic metamaterials will provide new vistas for applications where unidirectional transmission of energy is useful, e.g., for communication and sensing^3,7,9,20,33,34^, shock and vibration damping, and energy harvesting^13,17,21,35–37^. Our study opens up a plethora of future research directions, e.g., the investigation of odd elasticity^22^, instabilities^38^, and non-Hermitian physics^23–28,39^. Finally, we believe that recent developments in stimuli-responsive materials^40,41^ and robotics—via MEMS or graphene origami^19,42^—will allow to embed active control in materials as a smaller scale, for higher dimensions, and beyond rotational degrees of freedom—e.g., for acoustics, flexural waves, and quantum systems.

## Methods

### Mass-and-spring model with nonreciprocal springs

In this section, we describe the mass-and-spring model with nonreciprocal interactions discussed in Fig. 1 of the main text and derive its continuum limit (Eq. (1) of the main text). We then calculate its solutions in the frequency domain as well as its Green’s function.

Consider a mass-and-spring model, where all masses *m* are equal and all the springs are equivalent and of rest-length *p*. Newton’s second law reads

2 $$m\frac{{d^2u_j}}{{dt^2}} = F_{j - 1 \to j} + F_{j + 1 \to j},$$

where *u*_*j*_ is the displacement of mass *j* and *F*_*j*−1→*j*_ = *k*_*j*−1→*j*_(*u*_*j*−1_ − *u*_*j*_) (*F*_*j*+1→*j*_ = *k*_*j*+1→*j*_(*u*_*j*+1_−*u*_*j*_)) is the force exerted by the spring between masses *j*−1 (*j* + 1) and *j*. In an ordinary reciprocal system, *k*_*j−1*→*j*_ = *k*_*j*→*j*−1_ = *k*, and the right-hand side of Eq. (2) becomes *k*(*u*_*j*−1_ + *uj* − 2*u*_*j*_). By contrast, here we consider a special case where the springs are nonreciprocal $k_{j - 1 \to j} = k(1 + \varepsilon ) \ \ne \ k_{j \to j - 1} = k(1 - \varepsilon )$. In such a model, Newton’s action–reaction third law is broken, which means that in practice one needs to add local momentum at each site *j* to realize such a system. The equation of motion for mass *j* becomes

3 $$m\frac{{d^2u_j}}{{dt^2}} + k(1 + \varepsilon )(u_j - u_{j - 1}) + k(1 - \varepsilon )(u_j - u_{j + 1}) = 0.$$

In order to study the behavior of such a system, it is useful to consider the continuum limit, given by *u*_*j*_ → *u*(*x*) and *u*_*j*±1_ → *u*(*x*) ± *p*(*du*/*dx*) + *p*^2^/2(*d*^2^*u*/*dx*^2^), and which then leads to Eq. (1) of the main text. In the case of a reciprocal system (*ε* = 0), Eq. (1) becomes the wave equation, which admits dispersion-free mechanical waves of group and phase velocity *c*. For arbitrary (*ε* ≠ 0), Eq. (1) is analogous to the Telegrapher’s equation^43,44^, but where time and space have been interchanged.

If we assume that the medium is infinite, the Floquet–Bloch theorem predicts that plane wave solutions of the form *u*(*x*, *t*) = *u*_0_ exp*i* (*ωt* − *qx*) are solutions of Eq. (1), where *ω* is the radial frequency and *q* the wave vector. By inserting this expression in Eq. (1), we obtain the following dispersion relation

4 $$- \frac{{\omega ^2}}{{c^2}} + q^2 - \frac{{2\varepsilon i}}{p}q = 0.$$

Therefore, for a given *ω*, solutions are of the form *u*(*x*, *t*) = exp(*iωt*)exp(−*iq*_±_*x*), where $q_ \pm = \frac{i}{p}\left( {\varepsilon \pm \sqrt {\varepsilon ^2 - \frac{{p^2\omega ^2}}{{c^2}}} } \right)$. This result is discussed in the main text.

We calculate the Green’s function of Eq. (1) by using Fourier–Laplace transforms (see Supplementary Note 1) and find

5 $$u(x,t) = \frac{{e^{\varepsilon x/p}}}{2}\Theta \left( {t - \frac{{|x|}}{c}} \right)\left( {J_0\left( {\varepsilon \frac{{\sqrt {c^2t^2 - |x|^2} }}{p}} \right) - \frac{{(ct - |x|)^2}}{{c^2t^2 - |x|^2}}J_2\left( {\varepsilon \frac{{\sqrt {c^2t^2 - |x|^2} }}{p}} \right)} \right),$$

where Θ is the Heaviside step function and where *J*_*k*_ are Bessel functions of the first kind. This solution predicts that a pulse in *x* = 0 at time *t* = 0 leads to an asymmetric step function propagating with an exponentially increasing (decreasing) amplitude (see Fig. 1c of the main text) for forward (backward) propagation. In addition, we can rationalize this behavior by restricting our attention to the moving frame, that is, |*x*| = *ct*, we find

6 $$u_{{\mathrm{movingframe}}}(t) = \left\{ {\begin{array}{*{20}{l}} {\frac{1}{2}{\mathrm{exp}}\varepsilon ct/p} \hfill & {{\mathrm{for}}\,x \ > \ 0} \hfill \\ {\frac{1}{2}{\mathrm{exp}} - \varepsilon ct/p} \hfill & {{\mathrm{for}}\,x \ < \ 0}, \hfill \end{array}} \right.$$

which is discussed in the main text. Note that in the limit *ε* → 0, Eq. (1) becomes the 1D wave equation. Our solution is consistent with this limit; since *J*_0_(0) = 1 and *J*_2_(0) = 0, Eq. (5) becomes the well-known Green’s function for the 1D wave equation^45^ $u(x,t) = \frac{1}{2}\Theta \left( {t - \frac{{|x|}}{c}} \right)$.

### Realization of the robotic mechanical metamaterial

The 1D robotic mechanical metamaterial shown in Fig. 2a of the main text consists of a chain of mechanically coupled oscillators, each of which is combined with a minimal robotic system. These robotic unit cells, of dimensions 54 mm × 54 mm × 90 mm, are exact duplicates of each other. In this section, we provide details about the mechanical, electromechanical, and software characteristics of these building blocks.

Each oscillator is made up of (i) a 3D printed arm (ABS, red part in Supplementary Fig. 2a); (ii) two stainless-steel bolts and four nuts (M6) fastened to each side of the arm; (iii) the shaft of a coreless DC motor (Motraxx CL1628, blue part in Supplementary Fig. 2a); (iv) a custom-made aluminum shaft extension connected to the disk of an optical angular encoder (Broadcom HEDR-55L2-BY09, yellow part in Supplementary Fig. 2a). The total moment of inertia *J* = 27.550 ± 0.08 μkg m^2^ and the damping coefficient *γ* = 31.16 ± 2.34 μN s m have been determined via independent calibration measurements.

The oscillators are mechanically coupled to each other by two soft elastic beams made of vinylpolysiloxane (Elite double 8, Young’s modulus *E* = 0.25 MPa). The elastic beams are pre-stretched by 30% to avoid buckling when arms extremities come closer to each other. The structure of the elastic beams, shown in Supplementary Fig. 2b, c, is laser cut from a 2-mm thin cast sheet. The empty squares of the elastic structure are used to attach the elastic beam to the square-shaped protuberances of the oscillator arms. The square shape prevents any rotation of the elastic structure around its connection. In between each square, the elastic beam has an elongated hexagonal shape, with a maximum thickness of 6 mm and a minimum thickness of 1 mm.

When the oscillators rotate, the elastic beams can stretch and contract, with the deformations localized at the thin necks. Since the squared connection does not allow any sliding of the elastic structure on the oscillator’s arm, the elastic beam also undergoes bending deformations localized at the think necks. We describe the effect of these deformations on the energy of the system as follows: when the rotations of neighboring oscillators *θ*_L_ and *θ*_R_ are symmetrical (*θ*_L_ = −*θ*_R_), the elastic links primarily stretch/contract, and when the deformation of the neighboring oscillators is antisymmetical (*θ*_L_ = *θ*_R_), the links primarily bend. The energy of these two deformation modes can be expressed as (*C*/2)(*θ*_L_ − *θ*_R_)^2^ for the symmetrical mode and (*C*′/2)(*θ*_L_ + *θ*_R_)^2^ for the antisymmetrical mode. The torsional stiffnesses associated with these two deformation modes *C* and *C*′ differ. We optimized the geometry such that *C*′ = *C* (Supplementary Fig. 2c), see below for calibration. Note that the geometry to guarantee that *C*′ = *C* introduces unavoidable spurious vibrational effects, which are hard to control and which effectively reduce the stiffness *C* at large frequencies (**>**3 Hz, see Supplementary Fig. 5d).

The control system is made up of (i) an angular sensor (Broadcom HEDR-55L2-BY09, yellow part in Supplementary Fig. 2a); (ii) a coreless DC motor (Motraxx CL1628, blue part in Supplementary Fig. 2a) embedded in a cylindrical heatsink (in dark gray in Supplementary Fig. 2a); (iii) a microcontroller (Arduino ATmega32U4, integrated circuit in Supplementary Fig. 2a); (iv) a UART connection receiving the angular position of the right unit cell and transmitting its own angular position to the left unit cell.

*Angular encoder*: The angular sensor measures the position of the oscillator with a precision of 14000 PPR (pulse per revolution), which translates into a precision of 4 × 10^−4^ rad.

*Microcontroller*: The microcontroller digitizes the encoder’s signal with an angular resolution of 4 × 10^−4^ rad at a rate of 100 Hz and collects the angular position of the unit cell’s right neighbor through a serial protocol (UART connection) at a rate of 100 Hz. From a 9-bit timer, the microcontroller also builds up a pulse-width modulation (PWM) signal, to control the motor. The microcontroller is integrated to a custom-made electronic board that ensures power conversion and wiring to the motor, encoder, and the neighboring unit cells. The firmware of the microcontroller is uploaded from an external computer by serial communication (USB port). Although the microcontroller is not required to be connected to an external computer to function, if required, data can also be sent to en external computer (rate 100 Hz) via the same serial communication.

*DC motor*: In order to apply a given torque, the motor is controlled by a PWM signal. The PWM signal is sent to the motor and controls the generated torque with a resolution of 0.008 mN m, up to a maximum torque of *τ* = 3.92 mN m. Note that this maximum explains the saturation of the effective stiffness *C*_R→L_ for large values of the feedback parameter *α* in Fig. 2d in the main text.

*Software*: The basic algorithm of the software is depicted in Fig. 2c of the main text and runs continuously at a rate of 100 Hz. Each microcontroller collects the instantaneous angular position measures of its own angular sensor *θ*_L_ as well as the instantaneous angular position *θ*_R_ of the right neighbor. From those two signals, the microcontroller is programmed to output a PWM signal proportional to *g*(*θ*_L_ − *θ*_R_), where the constant *g* is a tunable gain parameter. This signal drives the DC motor, which leads to a torque proportional to *g*(*θ*_L_ − *θ*_R_), see below for calibration.

### Calibration and measurements

In order to perform measurements, the robotic metamaterial is actuated by a servomotor (Hitec D930DW, see Supplementary Fig. 3) controlled via a microcontroller (Arduino Mega 2560), interfaced to an external computer. The servomotor can be attached on either side of the setup and is mechanically connected to one oscillator via two green elastic structures (rectangular shape, 6-mm thick, Elite double 32, Young’s modulus *E* = 1 MPa, resulting in a torsional stiffness 27.4 mN m/rad, see Supplementary Fig. 4). These two elastic beams are sufficiently soft to ensure free rotations of the servomotor and negligible friction.

The stiffness measurements shown in Fig. 2d of the main text have been performed on two building blocks. The configuration of the setup is sketched in Supplementary Fig. 4a (see also Supplementary Fig. 5a). The left (right) oscillator was attached to a load cell (Instron 2530-5 N, resolution of 0.005 N, sampling frequency of 500 Hz) at a fixed position *θ*_L_ = 0 (*θ*_R_ = 0), allowing us to measure the torque, *τ*_R→L_ (*τ*_L→R_). The right (left) oscillator was driven by the servomotor, imposing an oscillatory angular displacement *θ*_R_ (*θ*_L_) at the frequency 0.1 Hz and amplitude 0.13 rad.

As shown in Supplementary Fig. 5a–c, at small angles, the torque varies linearly with the angular position *τ*_R→L_ = *C*_0,R→L_*θ*_R_ and *τ*_L→R_ = *C*_0,L→R_*θ*_L_. Note that this is also verified for angles up to 0.35 rad (not shown here). We therefore perform a linear fit to the torque-angle curves to extract the values of *C*_0,R→L_ and *C*_0,L→R_. In the case *α* = 0, we find *C*_0,R→L_ = *C*_0,L→R_ = *C*_0_ = 22.4 ± 0.4 mN m/rad (Supplementary Fig. 5c). For *α* > 0, we find *C*_0,R→L_ < *C*_0,L→R_ (Supplementary Fig. 5c). By using the same protocol at different excitation frequencies, we verified that the stiffnesses *C*_0,R→L_ and *C*_0,L→R_ are different for *α* ≠ 0 for any actuation frequency (see Supplementary Fig. 5d). Note that internal vibrations start to occur at 3 Hz, which we suspect are responsible for the decrease of the measured stiffnesses as the frequency increases.

Since our calibration experiments combine symmetric and antisymmetric deformations, *C*_0_ is related to the stiffnesses *C* and *C*′ defined above as *C*_0_ = *C* − *C*′. Similarly, *C*_0,R→L_ = *C*_R→L_ − *C*′ and *C*_0,L→R_ = *C*_L→R_ − *C*′. We calibrated *C*′ independently by using a slightly different experimental configuration. To do this, we connected rigidly the two oscillators such that they were moving with the same angle *θ*_L_ = *θ*_R_ and measured the force applied on both oscillators (see Supplementary Fig. 4b). We obtained a torsional stiffness *C*′ = 1.71 ± 0.04 mN m/rad, and as a result, *C* = 24.1 ± 0.4 mN m/rad as well as *C*_R→L_ and *C*_L→R_ shown in Fig. 2d of the main text.

Finally, we calibrated the DC motor by fixing the position of the left oscillator *θ*_L_ = 0 and measuring the torque $\tau _{\mathrm{L}}^{\mathrm{M}}$ exerted by the DC motor in the absence of the elastic beams as a function of the position of the right oscillator *θ*_R_ (see Supplementary Fig. 4c). As expected, we find that $\tau _{\mathrm{L}}^{\mathrm{M}}$ vs. *θ*_R_ is linear with a negative slope (not shown here), and by using a linear fit, we determine the gain function **−***κ*(*g*) as a function of the feedback parameter *g* controlling the amplitude of the PWM signal. For convenience, we rewrite *κ*(*g*) = *Cf*(*α*), where *α* is a dimensionless feedback parameter, and *f*(*α*) a dimensionless gain function. We observe that *f*(*α*) ≃ *α* is linear in the region *α* < 1 and saturates for larger *α* (see Fig. 2d and main text).

During experiments, the robotic metamaterial was mechanically excited at different frequencies by using the servomotor. In order to remain within the resolution limit of the angular encoder, yet to remain in the linear regime, we used an excitation amplitude of 0.21 rad (0.11) for frequencies below (above) 3 Hz. In the specific case of left excitations of the nonreciprocal metamaterial, given the amplification of the signal, we used smaller excitation amplitudes of 0.04 rad. As a result, the oscillation amplitude was always between 4 × 10^−4^ and 0.25 rad, well in the linear regime. During experiments, we recorded the instantaneous angular positions *θ*_*j*_(*t*) of each oscillator *j* at a rate of 100 Hz.

The amplitude of oscillation $\bar \theta _j$ of each time series *θ*_*j*_(*t*) was extracted from these measurements by performing a Fourier series analysis on the signal collected from the microcontrollers $\bar \theta _j = \sqrt {a_j^2 + b_j^2}$. The parameters *a*_*j*_ and *b*_*j*_ are the first coefficients of the Fourier series $a_j = \frac{2}{P}{\int}_{t_0}^{t_0 + P} \theta _j(t){\mathrm{cos}}\left( {\frac{{2\pi t}}{P}} \right)dt$ and $b_j = \frac{2}{P}{\int}_{t_0}^{t_0 + P} \theta _j(t){\mathrm{sin}}\left( {\frac{{2\pi t}}{P}} \right)dt$, with *P* the period of the impinging signal and *t*_0_ the initial time for the integration. Each experiment was performed over a minimum of 10 periods for signals above 10^−2^ Hz and 5 periods for signals below 10^−2^ Hz. The time *t*_0_ was carefully chosen such that the oscillations were stationary.

The amplitudes of oscillation as a function of the unit cell position are shown for 2 different frequencies in Fig. 3a (*α* = 0) and Fig. 3b (*α* = 0.43) of the main text. For frequencies lower than 3 Hz, the amplitude of oscillation decays exponentially. To compare these exponential decays with the predicted modes of oscillation, we superimposed the responses to left and right excitations by computing the angular displacement field $\bar \theta _j^{{\mathrm{lf}}} = \sqrt {(a_j^{\mathrm{l}} + a_j^{\mathrm{r}})^2 + (b_j^{\mathrm{l}} + b_j^{\mathrm{r}})^2}$, where $a_j^{\mathrm{l}}$ and $b_j^{\mathrm{l}}$ are the Fourier coefficients for the left excitation and $a_j^{\mathrm{r}}$ and $b_j^{\mathrm{r}}$ are the Fourier coefficients for the right excitation. We then fitted a double exponential decay of the form $\bar \theta ^{{\mathrm{lf}}}(j) = D_1e^{d_1j} + D_2e^{d_2j}$, where *D*_1_, *d*_1_, *D*_2_, and *d*_2_ are fitting parameters. The two decay rates *d*_1_ and *c*_2_ are plotted as green dots for different frequencies of actuation in Fig. 3c (*α* = 0) and Fig. 3d (*α* = 0.43) of the main text.

The angular displacement fields were also used to quantify the transmission of the signal (Fig. 3ef of the main text). For a given actuation frequency, the transmission from one side of the robotic metamaterial to the other is expressed as $T_{{\mathrm{R}} - > {\mathrm{L}}}(f) = 20\,{\mathrm{log}}_{10}\left( {\frac{{\bar \theta _{\mathrm{L}}^{{\mathrm{out}}}}}{{\bar \theta _{\mathrm{R}}^{{\mathrm{in}}}}}} \right)$ and $T_{{\mathrm{L}} - > {\mathrm{R}}}(f) = 20\,{\mathrm{log}}_{10}\left( {\frac{{\bar \theta _{\mathrm{R}}^{{\mathrm{out}}}}}{{\bar \theta _{\mathrm{L}}^{{\mathrm{in}}}}}} \right),$ where $\bar \theta _{\mathrm{R}}^{{\mathrm{in}}}$ and $\bar \theta _{\mathrm{L}}^{{\mathrm{in}}}$ ($\bar \theta _{\mathrm{R}}^{{\mathrm{out}}}$ and $\bar \theta _{\mathrm{L}}^{{\mathrm{out}}}$) correspond to the input (output) amplitude of the signal on the right and left side of the metamaterial, respectively. For excitation frequencies below 3 Hz, the output displacement is close to the resolution of the angular encoders 4 × 10^−^^4^ rad. Instead of directly measuring the output amplitudes of oscillation, we deduced them from an exponential decay fit over all oscillator amplitudes $\bar \theta _j$.

### Numerical model of the robotic metamaterial

In this section, we derive a model that closely describes the response of the experimental implementation of our robotic mechanical metamaterial and solve it numerically. We model a robotic metamaterial consisting of *N*-coupled oscillators, with instantaneous angular displacement $\underline \theta = [\theta _1,...,\theta _N]$. As discussed above, we assume that the deformations of the elastic rubber bands have both symmetric and antisymmetric components, whose potential energy reads respectively $V_{\mathrm{s}} = (C/2)\mathop {\sum}\nolimits_{j = 1}^{N - 1} (\theta _j - \theta _{j + 1})^2$ and $V_{\mathrm{a}} = (C{\prime}/2)\mathop {\sum}\nolimits_{j = 1}^{N - 1} (\theta _j + \theta _{j + 1})^2$. Therefore, the resulting elastic torque on oscillator *j* is *τ*_E_ = −∂(*V*_s_ + *V*_a_)/∂*θ*_*j*_. In addition, we model dissipation as an angular velocity-dependent term, $\tau _{\mathrm{D}} = - \gamma \dot \theta _j$, where *γ* is the damping coefficient. Finally, we assume that the additional feedback torque exerted by the DC motors is of the form *τ*_M_ = *Cα*(*θ*_*j*_ − *θ*_*j*+1_), where *α* is the dimensionless feedback parameter. Altogether, assuming that each oscillator has a rotational moment of inertia *J*, the equation of motion for the robotic metamaterial reads

7 $$\left[ {\begin{array}{*{20}{c}} \dot{\underline \theta } \\ \ddot{\underline \theta } \end{array}} \right] + \underline {\underline L } \left[ {\begin{array}{*{20}{c}} {\underline \theta } \\ \dot{\underline \theta } \end{array}} \right] = 0,$$

where $\underline {\underline L }$ is the 2*N* × 2*N* matrix

8 $$\underline {\underline L } = \left[ {\begin{array}{*{20}{c}} {\underline {\underline 0 } } & { - \underline {\underline I } } \\ {\frac{1}{J}\underline {\underline K } } & {\frac{\gamma }{J}\underline {\underline I } } \end{array}} \right].$$

where $\underline {\underline 0 }$ is the *N* × *N* zero matrix, $\underline {\underline I }$ is the *N* × *N* identity matrix, and $\underline {\underline K }$ is the matrix

9 $$	\underline {\underline K } = \\ 	\left[ {\begin{array}{*{20}{c}} {C(1 - \alpha ) + C{\prime}} & { - C(1 - \alpha ) + C{\prime}} & {} & {} & {} & {} \\ { - C + C{\prime}} & {C(2 - \alpha ) + 2C{\prime}} & { - C(1 - \alpha ) + C{\prime}} & {} & {} & 0 \\ {} & { - C + C{\prime}} & {C(2 - \alpha ) + 2C{\prime}} & { - C(1 - \alpha ) + C{\prime}} & {} & {} \\ {} & {} & \ddots & \ddots & \ddots & {} \\ 0 & {} & {} & { - C + C{\prime}} & {C(2 - \alpha ) + C{\prime}} & { - C(1 - \alpha ) + C{\prime}} \\ {} & {} & {} & {} & { - C + C{\prime}} & {C + C{\prime}} \end{array}} \right]_{N \times N}.$$

To solve for the dynamics upon excitations from the left (right) edge, we impose the angular displacement of the leftmost (rightmost) oscillator *θ*_1_ (*θ*_*N*_). The numerical data plotted in Figs. 3 and 4 of the main text correspond to the numerical solution of Eq. (8) by using the values of *C* = 24.1 mN m/rad, *C*′ = 1.71 mN m/rad, *J* = 27.55 μ kg m^2^, and *γ* = 31.16 μN m s calibrated above. Unless specified otherwise in the main text, we used *N* = 10 and *α* = 0 or *α* = 0.43. As for the boundary conditions, for Fig. 3, we have used a periodic forcing of the form $\theta _1 = \bar \theta _1e^{i\omega t}$ or $\theta _N = \bar \theta _Ne^{i\omega t}$, assumed stationary solutions of the form $\underline \theta = \underline {\bar \theta } e^{i\omega t}$, and solved the resulting algebraic equations numerically. For Fig. 4, the input excitations *θ*_1_ and *θ*_10_ are half-sine shape pulses of magnitude 0.04 rad and duration 100, which closely mimic a pulse excitation, and we have solved the system of ordinary differential numerically. We have analyzed the numerical data similarly to the experimental data (see above).

Eq. (7) is said to be linearly stable in time if the real part of all the eigenvalues of $- \underline {\underline L }$ is negative^46^. In Supplementary Fig. 6, we plotted the maximum of the real part of the eigenvalues of $- \underline {\underline L }$ as a function of the feedback parameter *α*. The graph shows that the system is stable (unstable) in time for *α* < *α*_c_ (*α* > *α*_c_) where *α*_c_ = 0.93 corresponds to an exceptional point. The precise value of *α*_c_ depends on *C*′/*C* and *γ*/*J* and converges to *α*_c_ = 1 for *C*′/*C* = 0 and *γ*/*J* = 0.

## Supplementary information

Supplementary Video 1

Supplementary Video 2

Supplementary Information

Description of Additional Supplementary Files

Peer Review File

Source Data

## Data Availability

The source data underlying Figs. 1–4 are provided as source data files. The data that support the plots within this paper and other findings of this study are available from the corresponding author upon request.

## Code availability

The codes that support the plots within this paper and other findings of this study are available from the corresponding author upon request.
