# Supplementary material for: Non-reciprocal robotic metamaterials
Source: Nat Commun. 2019 Oct 10;10:4608. doi: 10.1038/s41467-019-12599-3 (PMC6787071; doi:10.1038/s41467-019-12599-3)
Supplement: Supplementary file 3 — Supplementary Information [file 41467_2019_12599_MOESM3_ESM.pdf]

Supplementary information for Non-reciprocal robotic metamaterials

Martin Brandenbourger,[1] Xander Locsin,[1] Edan Lerner,[1] and Corentin Coulais[1,2]

[1]*Institute of Physics, Universiteit van Amsterdam,*
Science Park 904, 1098 XH Amsterdam, The Netherlands
[2]*Corresponding author : C.C., coulais@uva.nl*

SUPPLEMENTARY NOTE 1 : GREEN'S FUNCTION

We calculate the Green's function of the following Eq. (1) of the Main Text. To do this, we solve it with the right-hand side term $\delta(x)\delta(t)$, where δ is the Dirac Delta function. We first perform a Fourier transform in the spatial domain and the Laplace transform in the time domain defined as

$$\hat{u}(q,s) = \int_{-\infty}^{+\infty} dx \int_{0}^{+\infty} dt u(x,t) \exp(iqx - st) \tag{S1}$$

We find

$$\frac{1}{c^2} s^2 \hat{u}(q,s) + q^2 \hat{u}(q,s) - 2\varepsilon i q \hat{u}(q,s) = 1, \tag{S2}$$

where we have assumed that $p = 1$ without loss of generality and $u|_{t=0} = u_t|_{t=0} = 0$, $u|_{t=+\infty} = u_t|_{t=+\infty} = 0$ as well as $u|_{x=\pm\infty} = u_x|_{x=\pm\infty} = 0$. Eq. (S3) has a solution of the form

$$\hat{u}(q,s) = \frac{1}{\frac{s^2}{c^2} + q^2 - 2\varepsilon i q}. \tag{S3}$$

To find the solution in real space, we first invert the spatial Fourier transform

$$\bar{u}(x,s) = \frac{1}{2\pi} \int_{-\infty}^{\infty} dq \frac{e^{-iqx}}{\frac{s^2}{c^2} + q^2 - 2\varepsilon i q}, \tag{S4}$$

We calculate this integral by using a contour integral. The poles are at $q = i(\varepsilon \pm \sqrt{s^2/c^2 + \varepsilon^2})$. For $x > 0$ ($x < 0$), the integrand converges only if the contour is chosen in the lower (upper) half complex plane. We restrict our attention to $\varepsilon > 0$, therefore using Jordan's Lemma for contour integrals, the integral becomes

$$\bar{u}(x,s) = \begin{cases} -i\text{Res}\left(\frac{e^{-iqx}}{\frac{s^2}{c^2}+q^2-2\varepsilon i q}; q = i(\varepsilon - \sqrt{s^2/c^2+\varepsilon^2})\right) & \text{for } x > 0 \\ i\,\text{Res}\left(\frac{e^{-iqx}}{\frac{s^2}{c^2}+q^2-2\varepsilon i q}; q = i(\varepsilon + \sqrt{s^2/c^2+\varepsilon^2})\right) & \text{for } x < 0 \end{cases}, \tag{S5}$$

which leads to

$$\bar{u}(x,s) = \frac{e^{\varepsilon x}}{2} \frac{e^{-\frac{|x|}{c}\sqrt{s^2+c^2\varepsilon^2}}}{\sqrt{s^2+c^2\varepsilon^2}}. \tag{S6}$$

Then, we invert the Laplace transform

$$u(x,t) = \frac{e^{\varepsilon x}}{2} \frac{1}{2\pi i} \int_{\gamma - i\infty}^{\gamma + i\infty} ds \frac{e^{st - \frac{|x|}{c}\sqrt{s^2+c^2\varepsilon^2}}}{\sqrt{s^2+c^2\varepsilon^2}}, \tag{S7}$$

where γ is an arbitrary positive real number. We will again integrate this equation using contour integrals. For $t < |x|/c$, the integral is zero because there is no residue in the contour that converges. For $t > |x|/c$, we integrate using the contour $\mathcal{C}$ (See Supplementary Figure 1a), and find that

$$u(x,t) = \Theta\left(t - \frac{|x|}{c}\right) \frac{e^{\varepsilon x}}{4\pi i} \int_{\mathcal{C}} ds \frac{e^{st - \frac{|x|}{c}\sqrt{s^2+c^2\varepsilon^2}}}{\sqrt{s^2+c^2\varepsilon^2}}, \tag{S8}$$

where Θ is the Heaviside step function and the poles are $s = \pm ic\varepsilon$ are encircled in the contour $\mathcal{C}$. To solve that integral, we follow similar steps as in Goldstein [1] and perform the change of variables

$$s = c\frac{\varepsilon}{W}\left(\xi - \frac{W^2}{4\xi}\right), \tag{S9}$$

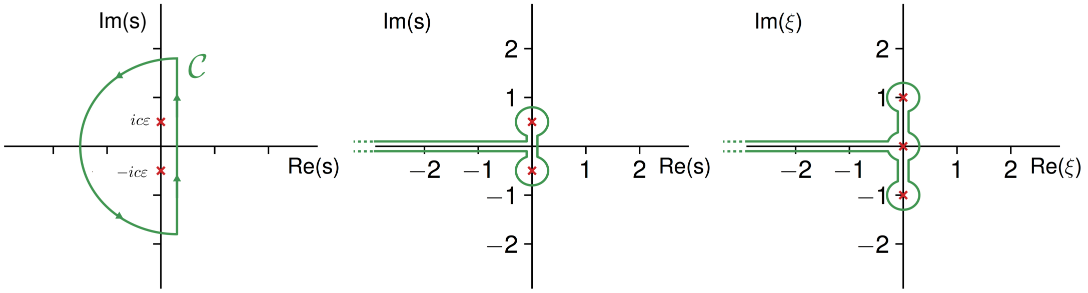

Supplementary Figure 1. Contours used to integrate Eq. (S8) (panels A and B) and Eq. (S10) (C).

where $W = \varepsilon(ct - |x|)$, which is necessarily positive. The Jacobian of the variable change is $J = \frac{\varepsilon}{W}\left(1+\frac{W^2}{4\xi^2}\right)$. Additionally, the integral does not change if we choose another contour, that encircles the poles, for instance $\mathcal{C}'$ (See Supplementary Figure 1b). Upon variable change, $\mathcal{C}'$ maps to $\mathcal{C}_\xi$ (See Supplementary Figure 1c), which encircles three poles at $\xi = 0$, $\xi = -iW/2$ and $\xi = iW/2$. In addition, note the identity $\sqrt{s^2 + c^2\varepsilon^2} = \frac{c\varepsilon\xi}{W}\left(1+\frac{W^2}{4\xi^2}\right)$. Therefore the new integral reads

$$u(x,t) = \frac{e^{\varepsilon x}}{2}\Theta\left(t - \frac{|x|}{c}\right)\frac{1}{2\pi i}\int_{\mathcal{C}_\xi}\frac{d\xi}{\xi}\frac{\left(1+\frac{W^2}{4\xi^2}\right)}{\left(1+\frac{W^2}{4\xi^2}\right)}e^{\xi - \frac{Y^2}{4\xi}}, \tag{S10}$$

where $Y = \varepsilon\sqrt{c^2t^2 - |x|^2}$ and where the contour $\mathcal{C}_\xi$ is depicted in Supplementary Figure 1c. Recall that the Bessel-Schläfli integrals definitions of the Lommel functions of two variables read (see Watson [2], section 16.58, p. 548)

$$U_0(W,Y) = \frac{1}{2\pi i}\int_{\mathcal{C}_\xi}\frac{d\xi}{\xi}\frac{1}{1+\frac{W^2}{4\xi^2}}e^{\left(\xi - \frac{Y^2}{4\xi}\right)}, \tag{S11}$$

$$U_2(W,Y) = \frac{1}{2\pi i}\int_{\mathcal{C}_\xi}\frac{d\xi}{\xi}\frac{\frac{W^2}{4\xi^2}}{1+\frac{W^2}{4\xi^2}}e^{\left(\xi - \frac{Y^2}{4\xi}\right)}, \tag{S12}$$

and that, alternatively (see Watson [2], section 16.5, p. 537 or Gradshteyn and Ryzhik [3], section 8.578, p. 987)

$$U_0(W,Y) = \frac{1}{2\pi i}\sum_{m=0}^{\infty}(-1)^m\left(\frac{W}{Y}\right)^{2m}J_{2m}(Y), \tag{S13}$$

$$U_2(W,Y) = \frac{1}{2\pi i}\sum_{m=0}^{\infty}(-1)^m\left(\frac{W}{Y}\right)^{2m+2}J_{2m+2}(Y), \tag{S14}$$

where J_k are Bessel functions of the first kind. Therefore the final solution is

$$u(x,t) = \frac{e^{\varepsilon x}}{2}\Theta\left(t - \frac{|x|}{c}\right)\left(J_0\left(\varepsilon\sqrt{c^2t^2 - |x|^2}\right) - \frac{(ct-|x|)^2}{c^2t^2 - |x|^2}J_2\left(\varepsilon\sqrt{c^2t^2 - |x|^2}\right)\right). \tag{S15}$$

For $p \neq 1$, ε can be substituted by ε/p in the previous equation and we obtain

$$u(x,t) = \frac{e^{\varepsilon x/p}}{2}\Theta\left(t - \frac{|x|}{c}\right)\left(J_0\left(\varepsilon\frac{\sqrt{c^2t^2 - |x|^2}}{p}\right) - \frac{(ct-|x|)^2}{c^2t^2 - |x|^2}J_2\left(\varepsilon\frac{\sqrt{c^2t^2 - |x|^2}}{p}\right)\right). \tag{S16}$$

SUPPLEMENTARY NOTE 2 : SUPPLEMENTARY FIGURES

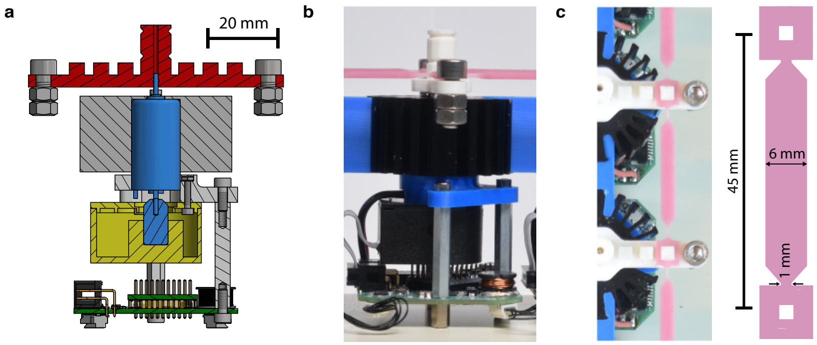

Supplementary Figure 2. (a) Computer-Aided Design (CAD) of one building block. The 3D printed arm used to mechanically connect each unit cell with elastic beams is shown in red. The motor and its shaft (both the inner shaft and the aluminium shaft extension) are shown in blue. They are surrounded by a heatsink (in dashed grey). The angular encoder is shown in yellow. The integrated circuit at the bottom (in green and black) depicts the microcontroller and the UART connections. The structural fixture of the unit cell is shown in light grey. (b) Photograph of one unit cell. The 3D printed arm (in white, now rotated by 90°) is connected with its neighbors by the elastic beams. (c) Close-up on one of the two elastic beams mounted on the robotic mechanical metamaterial and schematic of a part of a non-stretched beam.

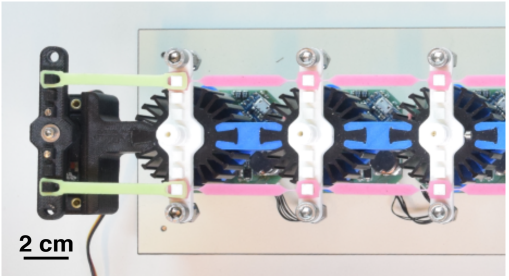

Supplementary Figure 3. Picture of the servomotor (Hitec D930DW) attached to the heatsink of one unit cell and mechanically connected to the left oscillator via two green rubber bands (rectangular shape, 6 mm thick, Elite double 32)

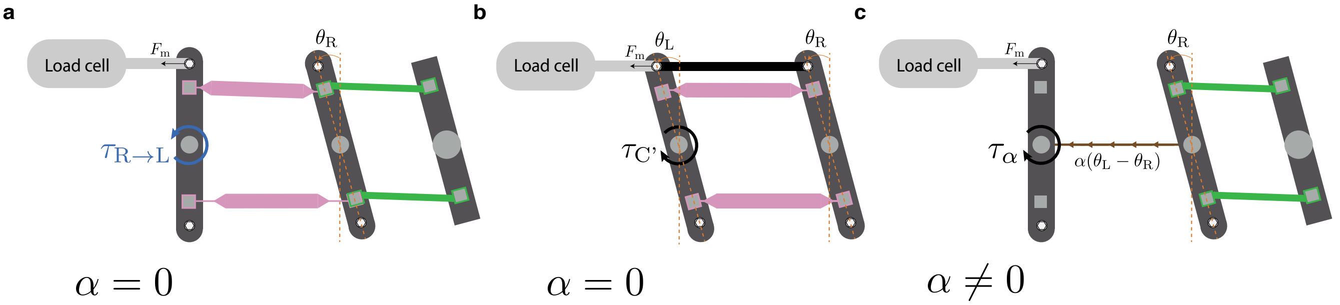

Supplementary Figure 4. Schematic of the torque and calibration measurements. (a) Calibration of the stiffness C via the measurement of the torque due to the bending and the stretching of the elastic beams as a function of the displacement of one unit cell. (b) Calibration of the stiffness C' via the measurement of the torque due to the bending of the elastic beam as a function of the displacement of both other unit cells ($\theta_L = \theta_R$). (c) Calibration of the feedback loop via the measurement of the torque applied by the servomotor as a function of the displacement of one unit cell.

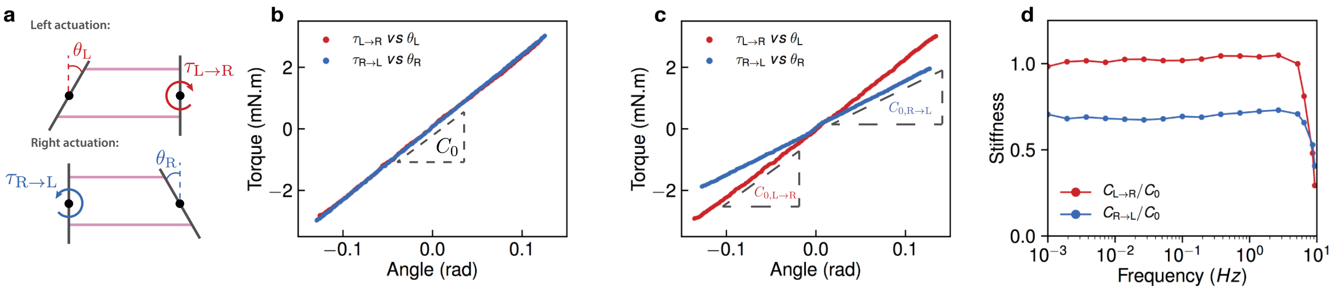

Supplementary Figure 5. Stiffness measurements. (a) Sketch of the (ab) Output torque measurement as a function of the angular displacement on two unit cells for $\alpha = 0$ (a) and $\alpha = 0.43$ (b) for left (red) and (right) actuations. (c) Stiffness deduced from the slope of the torque measurements as a function of the frequency of actuation for left (red) and right (blue) actuations.

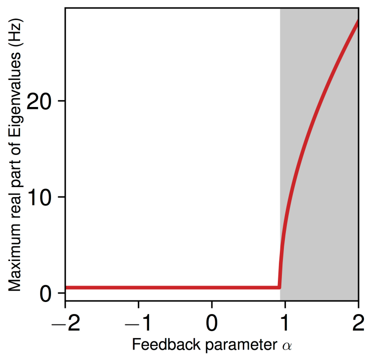

Supplementary Figure 6. Maximum of the real part of the eigenvalues of the matrix $-\underline{\underline{L}}$ as a function of the feedback parameter α. The system is unstable when at least one eigenvalue has a positive real part, namely for $\alpha > 0.93$ (grey shaded area).

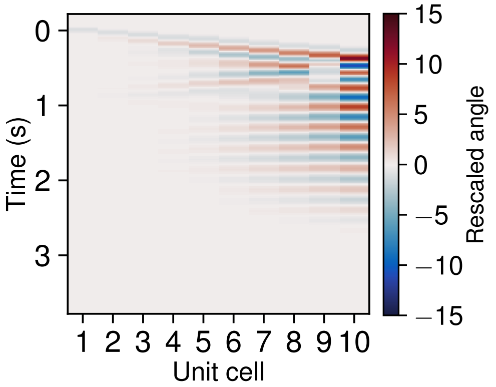

Supplementary Figure 7. Contour plots of the angular displacement vs. space coordinate and time for a feedback parameter $\alpha = 0.62$, upon pulse excitation on the left edge of the metamaterial (Extended time frame of Fig. 4d of the Main Text).

SUPPLEMENTARY REFERENCES

[1] S. Goldstein, On diffusion by discontinuous movements, and on the telegraph equation. *Quart. J. Mech. Appl. Math.* **17**, 129-156 (1951).

[2] G.N. Watson, "Neumann series and Lommel's functions of two variables" in *A Treatise on the Theory of Bessel Functions* (Cambridge University Press, Cambridge, UK, 1922).

[3] I. S. Gradshteyn, I. M. Ryzhik, Ed., *Table of integrals, series and products corrected and enlarged edition* (Academic Press, Cambridge, MA, 1980).

[4] K. Watanabe, "Integral Transform Techniques for Green's Function" in *Applied and Computational Mechanics 71* (Springer, Switzerland, 2014).
