# Supplementary material for: Non-reciprocal robotic metamaterials
Source: Nat Commun. 2019 Oct 10;10:4608. doi: 10.1038/s41467-019-12599-3 (PMC6787071; doi:10.1038/s41467-019-12599-3)
Supplement: Supplementary file 4 — Description of Additional Supplementary Files [file 41467_2019_12599_MOESM4_ESM.pdf]

Title: Supplementary Video 1:
Description: This Supplementary Video shows the robotic mechanical metamaterial excited on its left and right edge by harmonic excitations of the servomotor. The first (second) series of four videos shows the standing waves induced at 1.0 Hz with an amplitude of 0.21 rad and at 4.2 Hz with an amplitude of 0.11 rad and feedback parameter \alpha=0 (\alpha=0.43).

Title: Supplementary Video 2:
Description: This Supplementary Video shows the robotic mechanical metamaterial excited on its left and right edge by a pulse from the servomotor. The first (second) series of two videos shows the propagating waves induced by a pulse of duration 100 ms, amplitude 0.04 rad and feedback parameter \alpha=0 (\alpha=0.62). The last part of the video shows manual excitation at the fifth unit cell.
