# Supplementary material for: Non-reciprocal robotic metamaterials
Source: Nat Commun. 2019 Oct 10;10:4608. doi: 10.1038/s41467-019-12599-3 (PMC6787071; doi:10.1038/s41467-019-12599-3)
Supplement: Supplementary file 5 — Peer Review File [file 41467_2019_12599_MOESM5_ESM.pdf]

Editorial Note: This manuscript has been previously reviewed at another journal that is not operating a transparent peer review scheme. This document only contains reviewer comments and rebuttal letters for versions considered at *Nature Communications*. Mentions of the other journal have been redacted.

Reviewers' Comments:

Reviewer #1:
Remarks to the Author:
All my comments have been satisfactorily addressed and this paper is ready for publication.
